# Transcriptome Analysis of Two *Vicia sativa* Subspecies: Mining Molecular Markers to Enhance Genomic Resources for Vetch Improvement

**DOI:** 10.3390/genes6041164

**Published:** 2015-11-02

**Authors:** Tae-Sung Kim, Sebastin Raveendar, Sundan Suresh, Gi-An Lee, Jung-Ro Lee, Joon-Hyeong Cho, Sok-Young Lee, Kyung-Ho Ma, Gyu-Taek Cho, Jong-Wook Chung

**Affiliations:** 1 Department of Plant Resources, College of Industrial Science, Kongju National University, Yesan 340-702, Korea; E-Mail: tk227gm@gmail.com; 2 National Agrobiodiversity Centre, National Academy of Agricultural Science, Rural Development Administration, Jeonju 560-500, Korea; E-Mails: raveendars@gmail.com (S.R.); sureshplant@gmail.com (S.S.); gkntl1@korea.kr (G.-A.L.); jrmail@korea.kr (J.-R.L.); lsy007@korea.kr (S.-Y.L.); khma@korea.kr (K.-H.M.); 3 Department of Botany, Directorate of Distance Education, Madurai Kamaraj University, Madurai 625 021, India; 4 Department of Biological and Environmental Science, Dongguk University, Seoul 100-175, Korea; E-Mail: jrmail@korea.kr

**Keywords:** 454 pyrosequencing, the vetch (*Vicia sativa*), transcriptome, SSRs, SNPs

## Abstract

The vetch (*Vicia sativa*) is one of the most important annual forage legumes globally due to its multiple uses and high nutritional content. Despite these agronomical benefits, many drawbacks, including cyano-alanine toxin, has reduced the agronomic value of vetch varieties. Here, we used 454 technology to sequence the two *V. sativa* subspecies (ssp. *sativa* and ssp. *nigra*) to enrich functional information and genetic marker resources for the vetch research community. A total of 86,532 and 47,103 reads produced 35,202 and 18,808 unigenes with average lengths of 735 and 601 bp for *V. sativa sativa* and *V. sativa nigra*, respectively. Gene Ontology annotations and the cluster of orthologous gene classes were used to annotate the function of the *Vicia* transcriptomes. The *Vicia* transcriptome sequences were then mined for simple sequence repeat (SSR) and single nucleotide polymorphism (SNP) markers. About 13% and 3% of the *Vicia* unigenes contained the putative SSR and SNP sequences, respectively. Among those SSRs, 100 were chosen for the validation and the polymorphism test using the *Vicia* germplasm set. Thus, our approach takes advantage of the utility of transcriptomic data to expedite a vetch breeding program.

## 1. Introduction

The legume family (Fabaceae) is the third-largest family of flowering plants and the second most important plant family in agriculture [1]. Their N_2_-fixation capacity through mutualistic interactions with rhizobial soil bacteria and the resulting products, such as food, fodder, oil and fiber, make legumes a valuable resource [2,3]. “Green manuring” refers to incorporation of the soil of any field or forage crop while the crops are green or soon after flowering [4]. This procedure adds nutrients and organic matter to the soil, which prevents soil erosion, and helps suppress weeds, insect pests and disease, as well. Many legumes are used for this purpose, because they supply substantial amounts of N to the subsequent crop through N_2_ fixation [2,4,5], which offers an economically-attractive and ecologically-sound means of reducing external input and increasing sustainability [4,5]. *Vicia sativa*, known as the common vetch (hereafter, vetch), is one of the most commonly-grown winter cover crops [6]. It is also used as pasture, silage and hay. Vetch provides both cool-weather weed suppression and prevents fall N scavenging in mixtures with cereal grains. Furthermore, vetch has been applied successfully to vineyards and orchards. Thus, due to its economic and ecological advantages, vetch is now widespread throughout many parts of world, including the Mediterranean Basin, west and central Asia, China, eastern Asia, India and the USA [6,7].

Seeds of vetch are quite similar to those of lentils and are highly nutritional [6,7,8,9,10]. However, due to the cyano-alanine toxin in the seed, its use as a feed or food source is tightly restricted [7,11,12]. However, the lack of genomic resources in the public domain has hampered the related breeding programs to improve vetch [13].

Legumes are the targets of extensive sequence-based genomics research. Notably, completed and annotated genomes of the three legume species, *Glycine max* (soybean), *Medicago truncatula* and *Lotus japonicus*, have become available [1]. These reference genomic sequences have provided an opportunity to increase our understanding of the functions of genes associated with biologically-important traits in legumes [1]. Despite the usefulness of these reference genomic resources, in general, specific resources are required for the legume plants, including Vicia species, in which the relevant sequence or genomic information is not yet available [3].

In this context, transcriptome analysis using 454 pyrosequencing offers a powerful platform to meet this need [14]. Next generation sequencing (NGS) technology is a high-throughput, relatively cost-effective method of generating large-scale transcript sequences, which facilitate subsequent gene expression profiling and genome annotation [14]. Thus, the NGS approach provides new perspectives into the temporal and spatial regulation of genes that directly impact agronomic traits under various conditions, even in minor crop plants [15]. Furthermore, as the transcriptome itself can provide a precious resource for molecular markers, such as simple sequence repeats (SSRs) and single nucleotide polymorphisms (SNPs), this strategy could accelerate a breeding program by integrating genetic and functional information regarding agronomically-important genes [16].

In the previous study, we developed and characterized 65 novel polymorphic cDNA-SSR markers based on *V. sativa* transcriptome sequences [17]. Here, we further describe the development of *de novo* assembly and gene annotation of transcriptome datasets derived from cDNA samples obtained from two *V. sativa* subspecies. Two subspecies of *Vicia sativa*, *Vicia sativa* subsp. *sativa* (hereafter, sativa) and *Vicia sativa* subsp. *nigra* (hereafter, nigra), were selected. The sativa is currently the most popular vetch variety globally. While sativa grows well only under favorable conditions, nigra survives in diverse soils and climates, such as rocky slopes and meadows [13,18]. After the sequence assembly, BLAST searches were carried out against *Arabidopsis* and other sequence databases to infer gene functions using each unigene set from the 454 sequencing. We also searched potential SSR and SNP loci from unigene sequences and integrated those with putative functions. Using 100 randomly-selected SSRs, we further validated that *Vicia* SSRs can be used as an informative marker system. The candidate transcripts were also assigned for critical steps in the areas of the cyano-alanine-toxin pathway. The genomic information provided by this study will be useful for the vetch community, where only very few genetic data are currently available.

## 2. Experimental Section

### 2.1. Plant Materials

Sativa and nigra seeds were germinated in a glasshouse, and the leaves of the young seedlings were processed to extract mRNA. Total RNA isolation was performed using a TRIzol RNA isolation protocol (modified by D. Francis from Edgar Huitema) and the RNeasy Plant Mini kit (Qiagen, Valencia, CA, USA) following the manufacturer’s manual. Young seedling leaves (100 mg) were placed in liquid nitrogen, ground into a powder and subjected to total RNA extraction. Total RNA density was determined using a Biospec-Nano spectrophotometer (Shimadzu, Kyoto, Japan) and agarose gel electrophoresis. mRNAs were purified with the PolyATract mRNA Isolation System (Promega, Madison, WI, USA). The purified products were used to synthesize the full-length cDNA using the ZAP-cDNA Synthesis kit (Stratagene, Santa Clara, CA, USA).

### 2.2. Library Preparation

The cDNA was fragmented by nebulization using an Agilent 2100 bioanalyzer (Waldbronn, Germany) with a mean fragment size of ~600 bp. Approximately 1 µg cDNA was used to generate a library for genome sequencing with an FLX Titanium analyzer (Roche, Mannheim, Germany). The cDNA fragment ends were blunted, and two short adapters were ligated to each end according to standard procedures [19]. The adapters provided priming sequences for amplification and sequencing of the sample library fragments. They also served as a sequencing key, which is a short sequence of four nucleotides used by the system software for base calling. The sequencing key also released the unbound strand of each fragment (with 5-adaptor A) following repair of any nicks in the double-stranded DNA library. The quality of the single-stranded template DNA fragment library was assessed using the 2100 bioanalyzer, and the library was quantitated, including functional quantitation, to determine the optimal amount to use as input for emulsion-based clonal amplification.

### 2.3. 454 Sequencing

Single effective copies of template species from the DNA library were hybridized to DNA capture beads [20]. The immobilized library was then re-suspended in an amplification solution, and the mixture was emulsified, followed by PCR amplification. After amplification, the DNA-carrying beads were recovered from the emulsion and enriched. The second strands of the amplification products were melted away, leaving the amplified single-stranded DNA library bound to the beads. The sequencing primer was then annealed to the immobilized amplified DNA templates. After amplification, a single DNA-carrying bead was placed into each well of a picotiter plate (PTP) device for the following sequencing process [21,22]. To assign an individual sequencing read to the correct sample with high confidence, the GS FLX data analysis software was applied. The sequence assembly was carried out after sequencing using GS *de novo* Assembler software to produce contigs and singletons. Within a contig, there may exist several contig variants, mainly due to splice variants. Thus, we counted those isotigs as different individual unigenes.

### 2.4. Functional Category Annotation

First, we inferred potential functions of genes expressed in sativa and nigra using the Gene Ontology TAIR tool [23]. GO terms were assigned to the set of unigenes that showed hits against the *Arabidopsis thaliana* database, using the “Gene Ontology at TAIR” tool. Additionally, a BLASTx search was performed against the TAIR databases (*v.* 10), [24] with an *E*-value threshold <10^−5^. To annotate the function of the *Vicia* unigenes more specifically, we performed cluster of orthologous group (COG) analysis [25], wherein we BLASTed *Vicia* unigenes against the COG database (cutoff, *E*^−5^). Finally, we BLASTed *Vicia* unigenes against the NCBI non-redundant and UniProt databases (with an arbitrary expectation value of *E*^−5^) to obtain more comprehensive annotation information from diverse organisms to assign candidate transcripts associated with the cyano-alanine toxin production. 

### 2.5. Simple Sequence Repeat Mining and Validation 

All unigene sequences from 454 sequencing were used to search for SSR motifs with the ARGOS (*v.* 1.46) program [26] with a default setting. We randomly chose 100 SSR loci from nigra and sativa independently and designed primers flanking those SSR loci for the following polymorphism test to evaluate the marker efficiency of *Vicia* SSRs. We prepared DNA from eight sativa or nigra accessions to use as a polymerase chain reaction (PCR) template. The parameters to design PCR primers flanking the SSR loci were as follows: length range, 18–23 nucleotides with 21 as optimum; PCR product size range, 100–400 bp; optimum annealing temperature, 55 °C; GC content 40%–60%, with 50% as the optimum. Forward primers were synthesized by adding the M13 sequence to incorporate the fluorescent tail through additional PCR amplification. PCR conditions included a hot start at 95 °C for 10 min, followed by 10 cycles at 94 °C for 30 s, 60–50 °C for 30 s and 72 °C for 30 s, followed by 25 cycles at 94 °C for 30 s, 50 °C for 30 s and 72 °C for 30 sand a final elongation step of 72 °C for 10 min. PCR products were separated and visualized using the QIAxcel Gel Electrophoresis System (Qiagen, Valencia, CA, USA). The amplification intensity of individual markers was determined using an ABI Prism 3100 Genetic Analyzer (Applied Biosystems, Foster City, CA, USA), according to the manufacturer’s instructions, after adding the ABI GeneScan LIZ500 size standard and amplification product sizes determined by GeneMapper^®^ v3.7 software (Applied Biosystems). Polymorphic index content (PIC) values were calculated as described previously using the size information of amplicons from the eight accessions [27].

### 2.6. SNPs Discovery

We aligned the individual reads using the genome sequencer (GS) Reference Mapper software (Roche) to define the SNPs. This software automatically computes the alignment of reads from 454 sequencing against a reference sequence (the sativa sequence). To pinpoint SNPs, two criteria were applied; one criterion is “all difference”, where an SNP is called when at least 2 reads differ either from the reference sequence or from other reads aligned at a specific location [28]. Furthermore, there must be at least two non-duplicate reads that show the difference, that have at least 5 bases on both sides of the difference and that have few other isolated sequence differences in the read [28]. The other is “high-confidence differences”, which is a more stringent method. The requirements are as follows: (1) there must be at least 3 non-duplicate reads with the difference; (2) there must be both forward and reverse reads showing the difference, unless there are at least 7 reads with quality scores over 20 (or 30 if the difference involves a 5-mer or higher); (3) however, in case the difference is a single-base overcall or undercall, the reads with the difference must form the consensus of the sequenced reads (*i.e.*, at that location, the overall consensus must differ from the reference).

## 3. Results

### 3.1. 454 Sequencing 

A summary of the 454-sequencing data and the following sequence assembly analyses for the *V. sativa* subspecies is presented in Table 1. Based on the GS FLX sequencer standard procedures, the transcriptome sequencing yielded 28.43 and 16.06 Mb from 86,532 (sativa) and 47,103 reads (nigra), respectively, generating 331 (sativa) and 342 bp (nigra) sequence lengths on average (calculated from the total number of reads of the total number of bases (Table 1A); see Supplementary Material, Figure S1, on the journal’s website). Raw data from the 454 sequencing run was submitted to the National Center for Biotechnology Information (NCBI) Short Read Archive (SRA) and can be retrieved as Accessions SRP044088 and SRP044089. The two sample reads were assembled separately by *de novo* Assembler (Table 1). A total of 42,405 of the sativa sequence reads were fully incorporated into the assembly, resulting in 2698 contigs or isotigs along with 34,938 singletons (Table 1B). For nigra, the 24,242 incorporated reads generated 837 isotigs and 19,646 singletons, respectively (Table 1A). To obtain valid singletons, we applied two subsequent cleaning processes. The first clean-up process was with SeqClean [29], which excluded various contaminants (*ex.* adaptor sequences, poly(A) tails, *etc*.) and low quality and low-complexity sequences. Then, the pre-screened singletons were processed by Lucy [30] to reassure confidence in addition to trimming vector sequences. As a result, most of the reads were valid singletons (90% of sativa and 91% of nigra) for assigning 31,504 (sativa) and 17,971 (nigra) singletons (Table 1B), resulting in 34,202 (sativa) and 18,808 (nigra) non-redundant sequences or unigenes (Table 1B).

Table 1Summary of *de novo* assembly of transcriptome sequences. Sizes of unigenes (**A**) and singletons (**B**) found in the *Vicia sativa nigra* and *sativa* transcriptomes.

**(A):** 

Sample	Large Contig (Length ≥100 bp)					Singletons after Sequence Cleanings (SeqClean, Lucy)	Total Valid Unigenes (Isotigs ^c^ + Singletons)
	Contigs	Bases	ACZ ^a^	N50 Contig Size ^b^	Largest Contig Size		
sativa	2698	1,983.375	735.13	782	3849	31,504	34,202
nigra	837	503,641	601.72	619	3345	17,971	18,808

**(B):** 

Sample (*V. sativa* spp.)	Total No. of Reads	Total No. of Bases	Assembled	Partial	Singleton	Repeat	Singletons after SeqClean	Singletons after Lucy
sativa	86,532	28,429.544	42,405	5923	34,938	24	31,744	31,504
nigra	47,103	16,060.539	24,242	2309	19,646	9	18,091	17,971

^a^ Average contig size; ^b^ contig size means that half of all bases reside in contigs of this size or longer; ^c^ isotig includes transcript variant mainly from alternative splicing.

### 3.2. Functional Classification of the *Vicia* Transcriptomes

**Figure 1:**
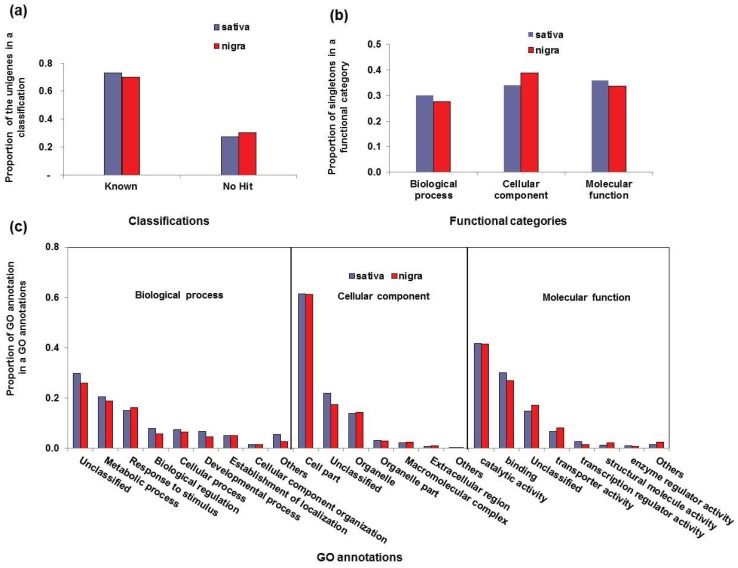
Summary of the functional annotation using the Gene Ontology (GO) approach for *Vicia sativa unigenes* by the classification (**a**); GO functional categories (**b**) and individual GO annotations (**c**) in between the *Vicia sativa nigra* and *sativa* transcriptome.

GO is able to annotate a putative gene function using a controlled vocabulary in terms of their associated biological processes, cellular components and molecular functions [31]. Thus, we utilized the GO assignments from *Arabidopsis* gene models to deduce the putative functions for the sativa and nigra unigenes (see Supplementary Material, Tables S1 and S2 on the journal’s website). Large numbers of the *Vicia* unigenes were assigned to GO categories (see Supplementary Material, Table S2 on the journal’s website), including about 75% (sativa) and 71% (nigra) of those, respectively (Figure 1a). Among them, 30% (sativa) and 28% (nigra) of GO annotated unigenes were assigned in the biological processes term; 34% (sativa) and 39% (nigra) as cellular components; 36% (sativa) and 34% (nigra) as molecular functions, respectively (Figure 1b and Table 2). We further specified those GO categories by a diverse set of putative functions (Figure 1c and Table 2). The abundant GO functions or terms between the two subspecies in each category were highly comparable, indicating that the contents and composition of the genes are similar to each other due to their genetic closeness. The most abundantly-assigned GO term in the biological process category, both for sativa and nigra, was metabolic processes (21% sativa and 19% nigra), followed by responses to stimuli (15% sativa, 16% nigra). Cell parts was the most abundant (62% sativa and 61% nigra) in the cellular component category, followed by organelles (14% sativa and 14% nigra); catalytic activity (42% sativa and 42% nigra) was the most abundant process in the molecular function category, followed by binding (30% sativa and 27% nigra) (Figure 1c and Table 2).

**Table 2:** Gene Ontology (GO) annotation results assigned to functional categories in the *Vicia sativa nigra* and *sativa* transcriptomes.

Functional Category	GO Annotations	Sativa		Nigra	
		No. of Unigenes	Proportion ^a^	No. of Unigenes	Proportion
Biological process	Unclassified	2194	0.29	1050	0.29
	Metabolic process	1433	0.19	749	0.19
	Response to stimulus	1055	0.14	703	0.18
	Biological regulation	597	0.08	235	0.06
	Cellular process	553	0.07	271	0.07
	Developmental process	503	0.07	188	0.05
	Establishment of localization	358	0.05	210	0.05
	Cell component organization	122	0.02	61	0.02
	Others	827	0.11	405	0.10
Cellular component	Cell part	5357	0.60	3227	0.62
	Unclassified	1797	0.20	902	0.17
	Organelle	1203	0.14	714	0.14
	Organelle part	278	0.03	161	0.03
	Macromolecular complex	189	0.02	128	0.02
	Extracellular region	73	0.01	61	0.01
	Others	10	0.00	46	0.01
Molecular function	Catalytic activity	3775	0.42	1836	0.41
	Binding	2757	0.30	1207	0.27
	Unclassified	1317	0.15	759	0.17
	Transporter activity	604	0.07	361	0.08
	Transcription regulator activity	239	0.03	70	0.02
	Structural molecule activity	146	0.02	111	0.02
	Enzyme regulator activity	98	0.01	34	0.01
	Others	148	0.02	114	0.03

^a^ No. of singletons in a GO annotation/total No. of singletons.

**Figure 2:**
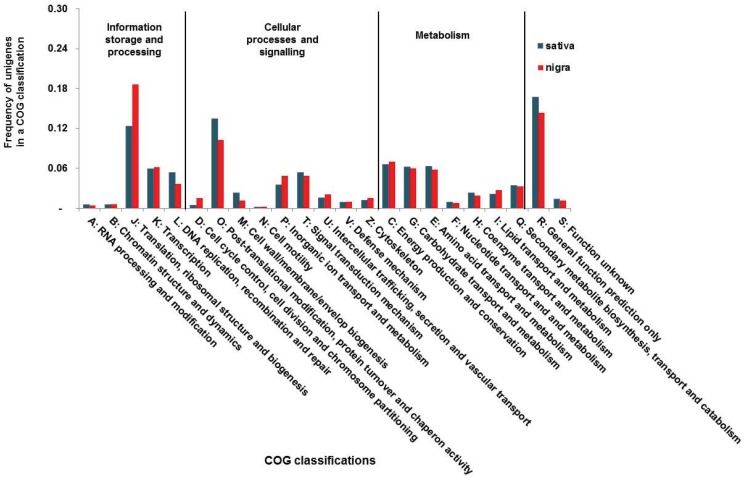
Cluster of orthologous groups (COG) classification between *Vicia sativa nigra* and *sativa* transcriptomes.

All unigenes were then subjected to BLASTs against the COG database to predict and classify more specific functions (see Supplementary Material, Tables S3 and S4 on the journal’s website). As the COG analysis requires translated protein sequences, quite fewer unigenes were functionally annotated and compared to those by the GO analysis. About 6% (sativa) and 2% (nigra) of each unigene set were functionally assigned by COG annotation (Table 2). Next, the annotated unigenes were functionally classified into at least 23 molecular families and three categories, including information storage and processing, cellular processes and signaling and metabolism (Figure 2). The frequencies of the functional sativa and nigra classes were similar, as shown the GO analysis (see Supplementary Material, Table S4 on the journal’s website). The most abundant functional classes were the J family (translation, ribosomal structure and biogenesis (12% sativa and 19% nigra)) in the information storage and processing category and the O family (post-translational modification and protein turnover chaperones; 13% sativa and 10% nigra) in the cellular processing and signaling category (Figure 2).

As the COG analysis requires translated protein sequences, which limits functional annotation of unigenes, we failed to find any putative functional homologs with regard to γ-GluBCA toxin production. In order to find putative functional homologs with regard to γ-GluBCA toxin production, we expanded it to the NCBI non-redundant and UniProt databases (method) (see Supplementary Material, Tables S5 and S6 on the journal’s website). As a result, we found candidate transcripts for key enzymes that catalyze the γ-GluBCA or detoxification pathways, including l-3-cyanoalanine synthase (isotig00826 of sativa, G7OXQHF01APLWW of nigra), γ-glutamyl transpeptidase (isotig02399, sativa) and β-cyano-l-alanine hydratase/nitrilase (isotig02627, sativa) (see Supplementary Material, Figure S2 and Table S7 on the journal’s website).

### 3.3. Simple Sequence Repeat Mining and Validation

SSR markers are tandemly repeated di-, *tri-* or *tetra-*nucleotide (hereafter, *di-*, *tri-*, *tetra-*nt) sequences [32]. It is one of the most popular molecular marker systems in plant breeding and genetics studies due to their highly polymorphic nature [32,33]. We used the ARGOS program [26] with default settings for the sativa and nigra unigenes to identify SSR markers in the *V. sativa* subspecies (Table 3). Overall, 4681 (sativa) and 2531 (nigra) SSRs were identified (Table 3). Although the absolute number of SSRs in the two subspecies appeared quite different, the frequencies were quite similar (Table 3), showing about 13% of the unigenes contained at least one SSRs in both cases. The SSR frequencies in *Vicia* species fell into the average group compared to those of other species, wherein 3%–20% of the unigenes or expressed sequence tags contain the putative SSRs [34,35,36,37].

**Figure 3:**
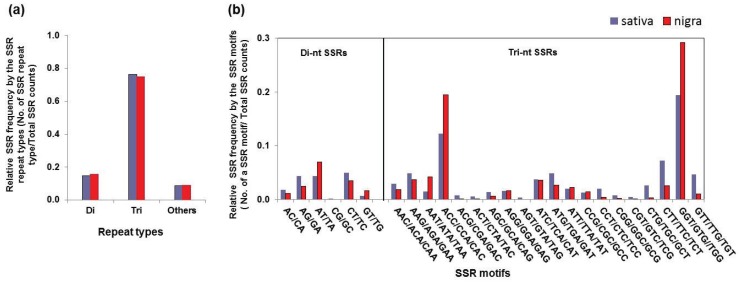
Frequency distribution of SSRs of *Vicia sativa nigra* and *sativa* transcriptomes, by the repeat types (**a**) and the motifs (**b**).

We subsequently compared the SSR frequencies by repeat type and motif. The frequencies by the repeat types were almost identical in sativa and nigra (Figure 3a). The major-class SSRs classified by repeat type belonged to *tri-*nt SSRs (76.3% sativa and 75.1% nigra), followed by *di-*nt SSRs (14.9% sativa and 15.7% nigra) (Figure 3a and Table 3). All other repeat types, such as *tetra-*, penta- and hexa-nucleotide motifs, were relatively low frequency (<10%). It was not surprising that *tri-*nt SSRs were dominant in the *Vicia* transcriptome. Otherwise, the SSRs may be under tight selection, as they may cause a frame shift, resulting in functional defects or a radical functional change in a gene if present in the coding sequence. Thus, it is thought that other SSR repeat types (except the *tri-*nt SSRs) are preferentially present only at the 5' or 3' untranslated regions. Considering that *di-*nt SSRs were the second most abundant in nigra and sativa, the *di-*nt SSR genes are presumably very active in the non-coding regions of *Vicia* transcripts.

**Table 3:** The distributions of simple sequence repeats (SSRs) identified from the *Vicia sativa nigra* and *sativa* transcriptomes.

Repeat Type	SSR Motif	Sativa		Nigra	
		Count	Frequency ^a^	Count	Frequency
*Di-*nucleotide	AC/CA	85	1.8%	27	1.1%
	AG/GA	205	4.4%	58	2.4%
	AT/TA	202	4.3%	172	7.0%
	CG/GC	4	0.1%	0	0.0%
	CT/TC	234	5.0%	86	3.5%
	GT/TG	31	0.7%	39	1.7%
	Subtotal	761	14.9%	398	15.7%
*Tri-*nucleotide	AAC/ACA/CAA	137	2.9%	46	1.8%
	AAG/AGA/GAA	228	4.9%	92	3.6%
	AAT/ATA/TAA	67	1.4%	105	4.1%
	ACC/CCA/CAC	573	12.2%	492	19.4%
	ACG/CGA/GAC	34	0.7%	3	0.1%
	ACT/CTA/TAC	26	0.6%	2	0.1%
	AGC/GCA/CAG	63	1.3%	14	0.6%
	AGG/GGA/GAG	76	1.6%	40	1.6%
	AGT/GTA/TAG	15	0.3%	0	0.0%
	ATC/TCA/CAT	173	3.7%	91	3.6%
	ATG/TGA/GAT	228	4.9%	68	2.7%
	ATT/TTA/TAT	94	2.0%	57	2.3%
	CCG/CGC/GCC	60	1.3%	35	1.4%
	CCT/CTC/TCC	95	2.0%	9	0.4%
	CGG/GGC/GCG	35	0.7%	6	0.2%
	CGT/GTC/TCG	22	0.5%	1	0.0%
	CTG/TGC/GCT	124	2.6%	8	0.3%
	CTT/TTC/TCT	338	7.2%	64	2.5%
	GGT/GTG//TGG	907	19.4%	739	29.2%
	GTT/TTG/TGT	220	4.7%	26	1.0%
	Subtotal	3515	76.3%	1898	75.1%
Other (Tetra/Penta/Hexa)	Subtotal	405	8.7%	235	9.3%
Total		4681		2531	

^a^ The proportion of a certain repeat type or motif out of total SSRs.

Furthermore, the proportions of each SSR motif in sativa and nigra were comparable (Figure 3b and Table 3). It has been reported that the occurrences of SSR motifs are unique or species specific, as they are influenced by the specific genomic context [27,32]. Therefore, the parallel frequencies of SSR motifs presumably represent a high degree of closeness between the sativa and nigra genomes. The most abundant SSR motif in the *tri-*nt SSRs was GGT/GTG//TGG (19.4% sativa and 29.2% nigra) followed by the ACC/CCA/CAC motif (12.2% sativa and 19.4% nigra) (Figure 3b and Table 3). Unlike the *tri-*SSRs, the most abundant SSR motif in the *di-*SSRs varied between the two subspecies (Figure 3a and Table 3); in nigra, both AT/TA and AG/GA motifs were enriched with a similar frequency, whereas AT/TA was dominant only in nigra (Figure 3b and Table 3). Thus, the occurrence of the SSR in the non-coding sequence might be more permissive than the coding region, resulting in *di-*ntSSR motifs of greater diversity.

**Figure 4:**
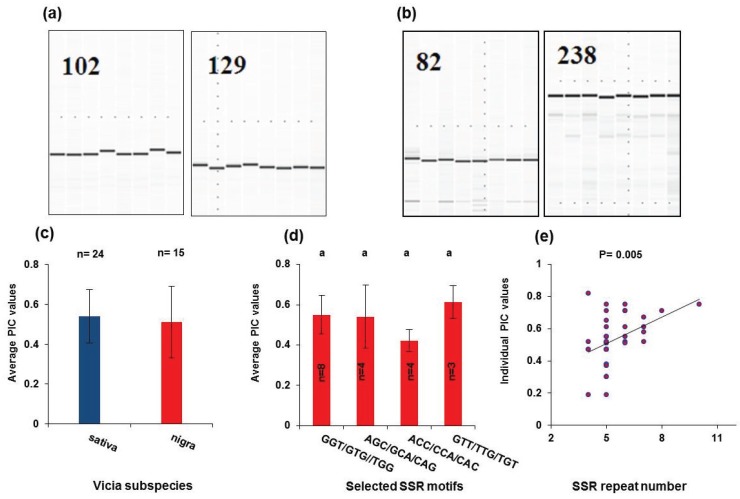
Empirical validations of the polymorphism of SSRs in *Vicia sativa nigra* and *sativa* using eight randomly-selected accessions. (**a**) Gel images showing the PCR products from the eight accessions for the representative SSR loci in *Vicia sativa sativa*; (**b**) those images for *Vicia sativa nigra*; (**c**) graph showing the overall average polymorphism index content (PIC) values; (**d**) average PICs by the SSR motif; (**e**) correlation between PIC and SSR repeat number. The numbers in gel images in (**a**,**b**) represent the names of the SSR loci; (**c**,**d**) n represents the number of SSR loci used to estimate PIC, and error bars indicate standard deviations; (**d**) different letters represent one(s) that is (are) significantly different from the others (determined by comparison of all pairs using the Tukey-Kramer HSD test); (**e**) *p*-values are determined by simple linear regression analysis.

We selected 100 primer pairs each from sativa and nigra and designed primers based on the flanking sequences (method) to estimate *Vicia* SSR marker efficiency. We also randomly selected eight accessions each from sativa and nigra and prepared DNA from those accessions to use as template for PCR analysis. We carried out the PIC analysis to estimate the degree of polymorphism in the sativa and nigra SSRs by using the size information of PCR products in each germplasm set (method). The PCR products harboring SSRs were amplified successfully at least in 23% (sativa) and 17% (nigra) of the tested SSR loci (Figure 4a,b; see Supplementary Material, Tables S8 and S9 on the journal’s website). We then carried out a PIC analysis using the PCR size information from the germplasm set (see Supplementary Material, Tables S8 and S9 on the journal’s website). The PIC values were ranged from 0.38 to 0.75 with an average of 0.54 (sativa) and 0.51 (nigra), respectively, indicating that *Vicia* SSRs are a potentially informative marker system (Figure 4c). We also estimated the polymorphism level of individual SSR motifs. Because the overall estimated PIC values between sativa and nigra were not significantly different within each other (*p* = 0.4746, one-way analysis of variance), we pooled the two datasets to determine more precise PIC estimates (Figure 4d). We found that the PIC estimates among individual *Vicia* SSR motifs were not significantly different among each other (from the comparison of all pairs using Tukey-Kramer HSD) (Figure 4d). However, a significant correlation was observed between the repeat number and the PIC value (simple linear regression analysis, *p* = 0.005), suggesting that repeat number may be an important parameter for the polymorphism (Figure 4e). Overall, *Vicia sativa* SSRs are thought to be informative, since the average PIC is more than 0.5 [27,32]. Thus, these SSR markers can be exploited to construct genetic linkage maps, identify genes and conduct parentage analyses in *Vicia* species.

### 3.4. SNPs Discovery

**Figure 5:**
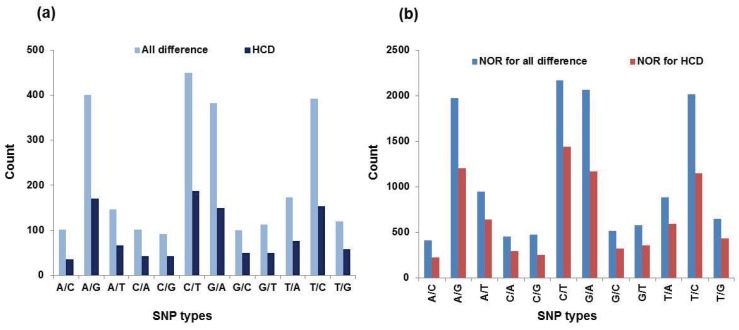
Distributions of single nucleotide polymorphism (SNPs) in *Vicia sativa nigra* and *sativa* transcriptomes by the types (**a**) and by the number of reads assembled in each SNP type (**b**).

We identified a total of 2571 candidate SNPs from 13,147 reads with the “all difference” criterion (method) (Figure 5 and Table 4). Of those, we added three more criteria to screen out the high confidence differences (HCD) in the sequences (method), although these requirements reduced sensitivity for detecting rare SNPs. As a result, 1080 SNPs were identified with high confidence out of 8104 reads (Figure 5 and Table 4). Thus, SNP density is estimated as 20% (with “all difference” and 13% (with “HCD”), respectively. Considering the ratio of unigenes to reads (0.3 based on the sativa transcriptome), about 4% of unigenes may harbor at least an “HCD” SNP. Within the detected SNP transition, 63% were much more common than those of transversion (37%) (Table 4). The proportions of A/G and C/T transitions were similar, as were the other four transversion types (A/T, A/C, G/T and C/G). Further studies are needed to investigate how informative or polymorphic the *Vicia* SNPs are (Table 4).

**Table 4:** Summary of SNPs discovered from the genome sequencer (GS) reference mapper.

SNP Types	All Differences		HCD ^a^		NOR ^b^ for All Differences		NOR for HCD	
	Number	Percentage ^c^	Number	Percentage	Number	Percentage	Number	Percentage
A/C	102	0.04	35	0.03	413	0.03	224	0.03
A/G	400	0.16	170	0.16	1974	0.15	1206	0.15
A/T	147	0.06	66	0.06	949	0.07	645	0.08
C/A	101	0.04	43	0.04	456	0.03	298	0.04
C/G	92	0.04	42	0.04	477	0.04	258	0.03
C/T	449	0.17	187	0.17	2168	0.16	1441	0.18
G/A	382	0.15	149	0.14	2064	0.16	1170	0.14
G/C	100	0.04	50	0.05	518	0.04	327	0.04
G/T	113	0.04	50	0.05	577	0.04	359	0.04
T/A	173	0.07	76	0.07	887	0.07	592	0.07
T/C	392	0.15	154	0.14	2016	0.15	1146	0.14
T/G	120	0.05	58	0.05	648	0.05	438	0.05
Total	2571	1.01	1080	1.00	13,147	0.99	8104	0.99

^a^ Highly-confident difference; ^b^ NOR, number of reads; ^c^ percentage of a certain SNP type in total SNPs.

## 4. Discussion

We used the 454 technology for transcriptome sequencing in the two *V. sativa* subspecies and recovered 28.43 and 16.06 Mb of nucleotide data from sativa and nigra, respectively, which further generated 86,532 and 47,103 clean reads. The clean reads yielded 34,202 and 18,808 unigenes with an average length of 735 and 601 bp for sativa and nigra, respectively (Table 1). These unigenes are very close to the estimated number of total genes (25,000) present in a typical diploid plant genome [17]. Furthermore, the average coding sequence length of 511 bp was reported in a vetch genome-scale gene expression study [23]. Moreover, the obtained results are comparable to those observed in other studies on: *Pisum sativum*, 454 bp [24]; *Pinus contorta*, 500 bp [38]; *Lens culinaris*, 770 bp [39]; *Ipomoea batatas*, 790 bp [30]; and *Vigna radiata*, 843 bp [40]. The remaining reads may have been the result of various reasons, such as the incompleteness of known databases, sequencing errors or short read lengths leading to a difficulty in assembly [39,41].

In regards to the functional clustering, large numbers of *Vicia* unigenes were functionally assigned by performing GO (about 70%) and COG (about 20%) analyses (Table 2), and the remaining unigenes could not be assigned to a specific functional annotation, either because they matched a protein of unknown function or because no homologous nucleotide sequence was found in the database. Lu *et al*. (2011), reported that many of the short sequencing reads cannot be matched to known genes during the identification of significant sequence similarity based on query sequence length [42]. Most unigenes could be clustered into the three main GO categories as biological process 30% (sativa) and 28% (nigra), cellular component 34% (sativa) and 39% (nigra) and molecular function 36% (sativa) and 34% (nigra) and further assigned to 23 COG functional classes. Hiremath *et al*. (2011) reported GO results of 20,634 (19.9%) tentative unique sequences (TUSs) were assigned to three principal categories: molecular function (10,963 TUSs), biological process (8099 TUSs) and cellular component (6662 TUSs) [40], and Gomes *et al.* (2012) reported that all identified proteins were distributed across 15 COG functional categories [43].

A total of 4681 (sativa) and 2531 (nigra) microsatellites were identified from 34,202 (sativa) and 18,808 (nigra) assembled unigenes, including *di-*, *tri-*, *tetra-*, penta- and hexa-nucleotide repeats. *Tri-*nucleotide repeats were the most common type in the cDNA-SSR dataset, with *di-* and other nucleotide repeats being present at much smaller frequencies. The characteristics of these microsatellites are summarized in Table 3. Previous research reported that the occurrence of SSR in the coding regions seems to be limited by non-perturbation of ORFs [44], and the *tri-* and hexa-nucleotide repeats are dominant in protein-coding exons of all taxa [45]. Besides, the relative proportions of EST-SSR motif types observed in this study coincided with previous reports, such as the vetch (*Vicia sativa*) [46], Ma bamboo (*Dendrocalamus latiflorus*) [47] and alfalfa (*Medicago sativa*) [48]. Furthermore, since we found that *Vicia* cDNA-SSRs are highly polymorphic, thus, those retrieved cDNA-SSR, through the NGS sequencing technology, will be a valuable resource for research communities of the vetch breeding program.

In the present study, we identified a total of 2571 candidate SNPs from 13,147 reads with the “all difference” criterion (Figure 5 and Table 4). Among the detected SNP transitions, 63% were much more common than those of transversion (37%) (Table 4). The proportions of A/G and C/T transitions were similar, as were the other four transversion types (A/T, A/C, G/T and C/G). Further studies are needed to investigate how informative or polymorphic the *Vicia* SNPs are (Table 4). Our results indicated that transitions were more numerous than transversions in terms of nucleotide substitutions. The work presented in [49] conducted a similar study on chickpea and identified 1022 SNPs that were classified as transitions or transversions based on nucleotide substitutions. The frequency of transitions and transversions was comparable to that observed in other plant species [50,51,52]. For the deep and redundant coverage produced over many genes, pyrosequencing of cDNA is ideal for SNP discovery and characterization [53,54,55].

The *Vicia* germplasms held at the International Center for Agricultural Research in the Dry Areas, Aleppo, Syria, have revealed that the cyano-alanine toxin level is not fixed, but rather varies quantitatively among accessions [7]. Moreover, a significant inverse association between seed size and toxin production was found in both nigra and sativa, although the magnitude is different [7]. The toxin content of small-seeded nigra seems to be more sensitively affected. Thus, this toxin can be diluted gradually with larger seed mass, implying that there may be a metabolic competition between the toxin production and seed development [7].

γ-GluBCA, the major form of cyano-alanine toxin in vetch, can be synthesized from l-3-cyanoalanine or beta-cyano-l-alanine, which originate from endogenous cyanide in the ethylene biosynthesis pathway (see Supplementary Material, Figure S2 on the journal’s website) [56,57,58,59]. Due to the limitation of COG analysis, we could not find any putative functional homologs with regard to γ-GluBCA toxin production. To exclude the possibility that the scope of our functional annotation was too narrow, we expanded it to the NCBI non-redundant and UniProt databases (method) (see Supplementary Material, Tables S5 and S6 on the journal’s website). As a result, we found candidate transcripts for key enzymes that catalyze the γ-GluBCA or detoxification pathways, including l-3-cyanoalanine synthase (isotig00826 of sativa, G7OXQHF01APLWW of nigra), γ-glutamyl transpeptidase (isotig02399, sativa) and β-cyano-l-alanine hydratase/nitrilase (isotig02627, sativa) (see Supplementary Material, Figure S2 and Table S7 on the journal’s website). Thus, these candidates can be used for future research, which aims to reduce cyano-alanine toxin levels in vetch.

Currently, the candidates found in sativa were not discovered in the nigra transcriptome, presumably due to the lower depth in the sequencing. Thus, more in-depth transcriptome analyses in vetch are needed to fill these gaps. Furthermore, it is possible that the genes controlling the toxin or detoxification may not be constitutively expressed, but rather regulated by factors, such as environmental stimuli or developmental controls. Thus, the temporal and spatial aspects of the toxin regulation, especially during seed production stages, should be considered in the future transcriptome investigations, so as to understand the molecular nature of cyano-alanine toxin production more thoroughly.

## 5. Conclusions

In conclusion, we have integrated all of the functional annotation information with the flanking primer sequence of SSRs in isotig sequences of sativa and nigra (see Supplementary Material, Tables S10 and S11 on the journal’s website). Since we found that *Vicia* cDNA-SSRs are highly polymorphic, thus, those retrieved cDNA-SSR, through the NGS sequencing technology, will be a valuable resource for research communities of the vetch breeding program, allowing identification of alleles that are either linked to or directly responsible for important traits, such as the cyano-alanine toxin production of the vetch seed.

## Supplementary Files

Supplementary File 1
