# Supplementary material for: Transcriptome Analysis of Two Vicia sativa Subspecies: Mining Molecular Markers to Enhance Genomic Resources for Vetch Improvement
Source: Genes (Basel). 2015 Nov 2;6(4):1164–82. doi: 10.3390/genes6041164 (PMC4690033; doi:10.3390/genes6041164)
Supplement: Supplementary File 1 [file genes-06-01164-s001.zip › Figure S2.pptx]

## Slide 1
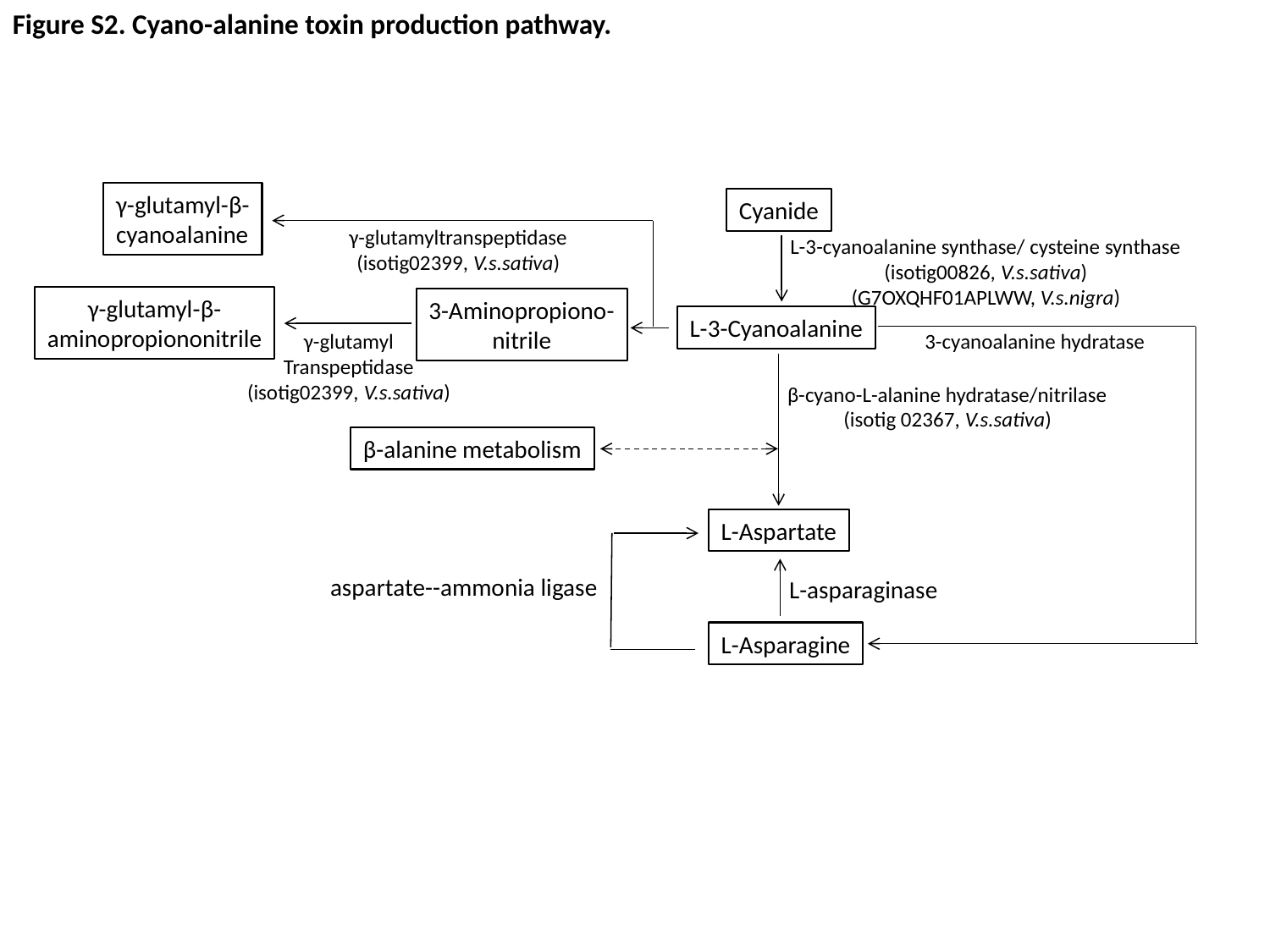

Figure S2. Cyano-alanine toxin production pathway.
γ-glutamyl-β-
cyanoalanine
Cyanide
γ-glutamyltranspeptidase
(isotig02399, V.s.sativa)
L-3-cyanoalanine synthase/ cysteine synthase
(isotig00826, V.s.sativa)
(G7OXQHF01APLWW, V.s.nigra)
γ-glutamyl-β-
aminopropiononitrile
3-Aminopropiono-
nitrile
L-3-Cyanoalanine
γ-glutamyl
Transpeptidase
(isotig02399, V.s.sativa)
3-cyanoalanine hydratase
β-cyano-L-alanine hydratase/nitrilase
(isotig 02367, V.s.sativa)
β-alanine metabolism
L-Aspartate
aspartate--ammonia ligase
L-asparaginase
L-Asparagine
