# Supplementary material for: Transcriptome Analysis of Two Vicia sativa Subspecies: Mining Molecular Markers to Enhance Genomic Resources for Vetch Improvement
Source: Genes (Basel). 2015 Nov 2;6(4):1164–82. doi: 10.3390/genes6041164 (PMC4690033; doi:10.3390/genes6041164)
Supplement: Supplementary File 1 [file genes-06-01164-s001.zip › genes-84097-Supplementary-publish.pdf]

Supplemental Materials

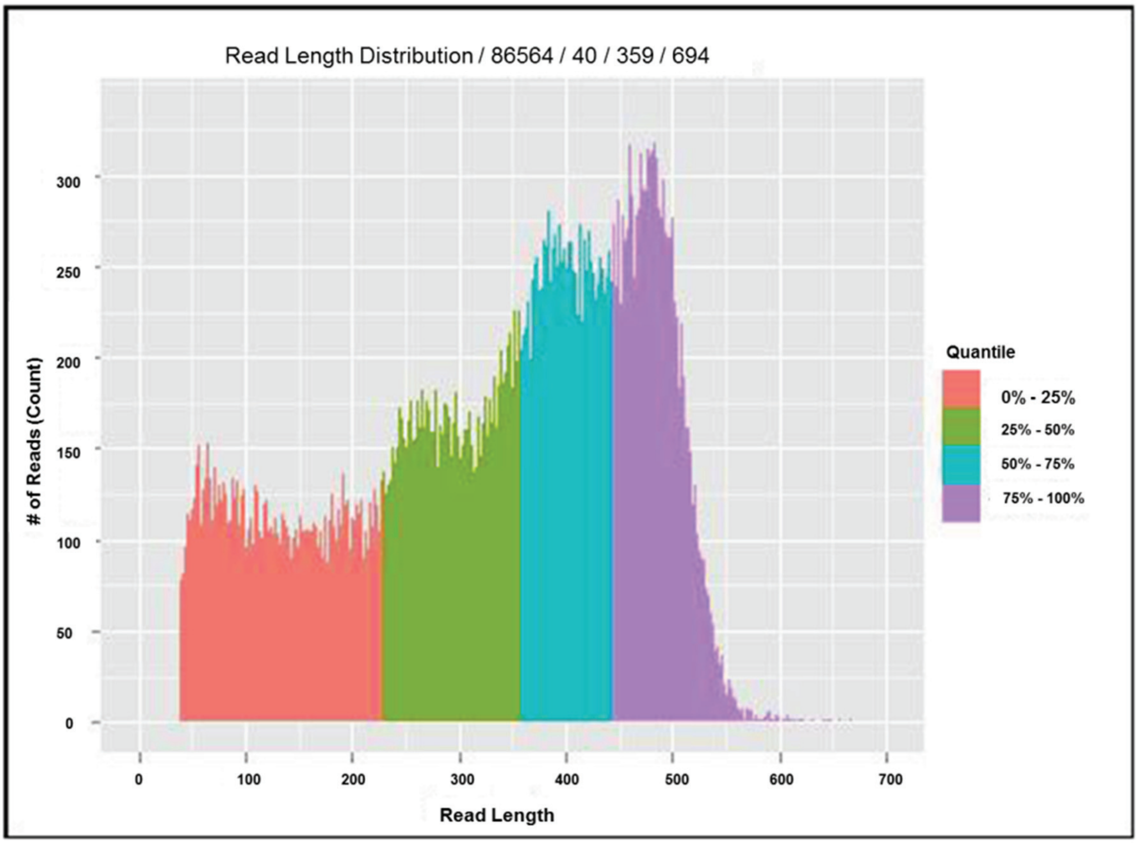

(**a**)

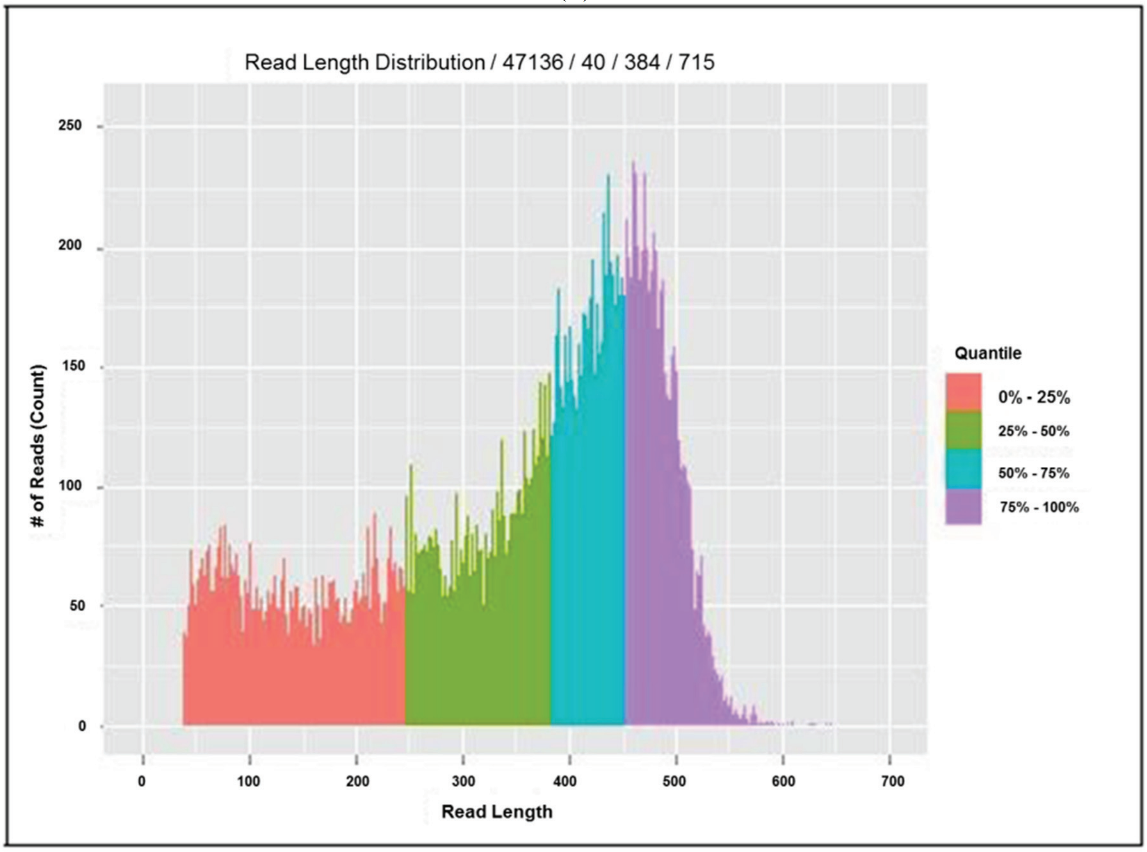

(**b**)

Figure S1. The read length distribution of the sativa (**a**) and nigra (**b**) transcriptome.

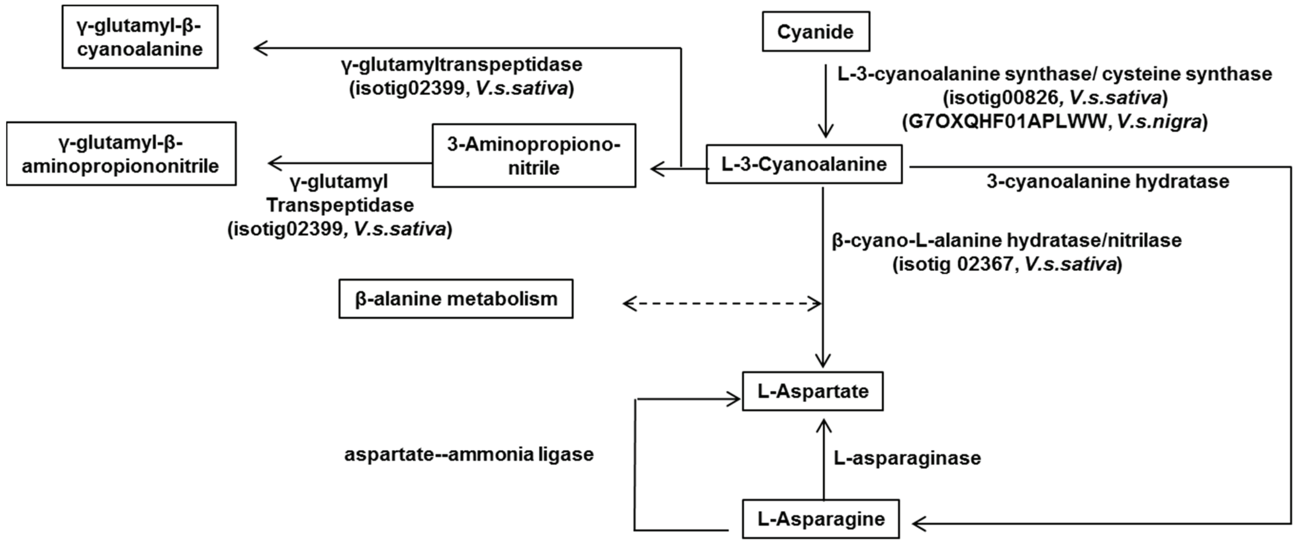

Figure S2. The cyano-alanine toxin production pathway.

This pathway information is modified from map00460 (cyanoamino acid metabolism) in the KEGG PATHWAY database [1].

Table S1. Results of functional annotations using the gene ontology (GO) approach in the individual *Vicia sativa sativa* unigenes.

Table S2. Results of functional annotations using the gene ontology (GO) approach in the individual *Vicia sativa nigra* unigenes.

Table S3. Results of functional annotations using cluster of orthologous groups (COG) analysis in the individual *Vicia sativa sativa* unigenes.

COG annotations were only applied to the unigenes wherein translated amino acids can be provided. More information for the functional letter and abbreviations for reference genomes in the data field for individual *Vicia sativa sativa* unigenes can be found at NCBI COG website [2].

Table S4. Results of functional annotations using cluster of orthologous groups (COG) analysis in the individual *Vicia sativa nigra* unigenes.

COG annotations were only applied to the unigenes wherein translated amino acids can be generated. More information for the functional letter and abbreviations for reference genomes in the data field for individual *Vicia sativa nigra* unigenes can be found at NCBI COG website [2].

Table S5. Results of functional annotations from the BLAST against NCBI non-redundant, and UniProt databases for the individual *Vicia sativa sativa* unigenes.

Table S6. Results of functional annotations from the BLAST against NCBI non-redundant, and UniProt databases for the individual *Vicia sativa nigra* unigenes.

Table S7. Summary of candidate transcripts associated in cyano-alanine toxin production pathway.

***V. sativa* spp.**	**ID**	**Length**	**Description**	**Locus**	**Score**	
					**Bit**	***E*-Value**
sativa	isotig02627	296	Putative nitrilase-associated protein (Arachis hypogaea)	ACF74272.1	107	6.00E−22
sativa	isotig02399	444	Putative gamma-glutamyl transferase (Populus tremula × Populus alba)	ABB59572.1	152	2.00E−35
sativa	isotig00826	985	Cysteine × synthase (Glycine max)	ABQ88337.1	542	2.00E−152
nigra	G7OXQHF01APLWW	454	Cysteine synthase (Glycine max)	ABQ88337.1	118	6.00E−57

Table S8. Primer information used for marker efficiencies of *Vicia sativa sativa* SSRs.

Marker names, forward and reverse primer sequences, SSR motifs, and numbers and sizes of alleles amplified from 24 randomly selected *Vicia sativa sativa* SSRs for 8 *Vicia sativa sativa* accessions are shown in the table.

Table S9. Primer information used for marker efficiencies of *Vicia sativa nigra* SSRs.

Marker names, forward and reverse primer sequences, SSR motifs, and numbers and sizes of alleles amplified from 24 randomly selected *Vicia sativa nigra* SSRs for 8 *Vicia sativa nigra* accessions are shown in the table.

Table S10. Integrated results of functional annotations with potential SNP and SSR for individual *Vicia sativa sativa* isotigs.

Functional annotations from our study including GO, COG and the BLAST against NCBI non-redundant, and UniProt databases for the individual isotigs of *Vicia sativa sativa* are integrated. In addition, flanking primer sequences of SSRs along with expected product sizes and melting temperature (Tm) in the corresponding isotigs are described.

Table S11. Integrated results of functional annotations with potential SNP and SSR for individual *Vicia sativa nigra* isotigs.

Functional annotations from our study including GO, COG and the BLAST against NCBI non-redundant, and UniProt databases for the individual isotigs of *Vicia sativa nigra* are integrated. In addition, flanking primer sequences of SSRs along with expected product product sizes and melting temperature (Tm) in the corresponding isotigs are described.

References

1. KEGG PATHWAY Database. Avaliable online: www.genome.jp/kegg/pathway.html (accessed on 20 September 2015).

2. Phylogenetic Classification of Proteins Encoded in Complete Genomes. Avaliable online: http://www.ncbi.nlm.nih.gov/COG/ (accessed on 20 September 2015).

© 2015 by the authors; licensee MDPI, Basel, Switzerland. This article is an open access article distributed under the terms and conditions of the Creative Commons Attribution license (http://creativecommons.org/licenses/by/4.0/).
