# Supplementary figures and images for: Transcriptome Analysis of Two Vicia sativa Subspecies: Mining Molecular Markers to Enhance Genomic Resources for Vetch Improvement
Source: Genes (Basel). 2015 Nov 2;6(4):1164–82. doi: 10.3390/genes6041164 (PMC4690033; doi:10.3390/genes6041164)

## Slide 1
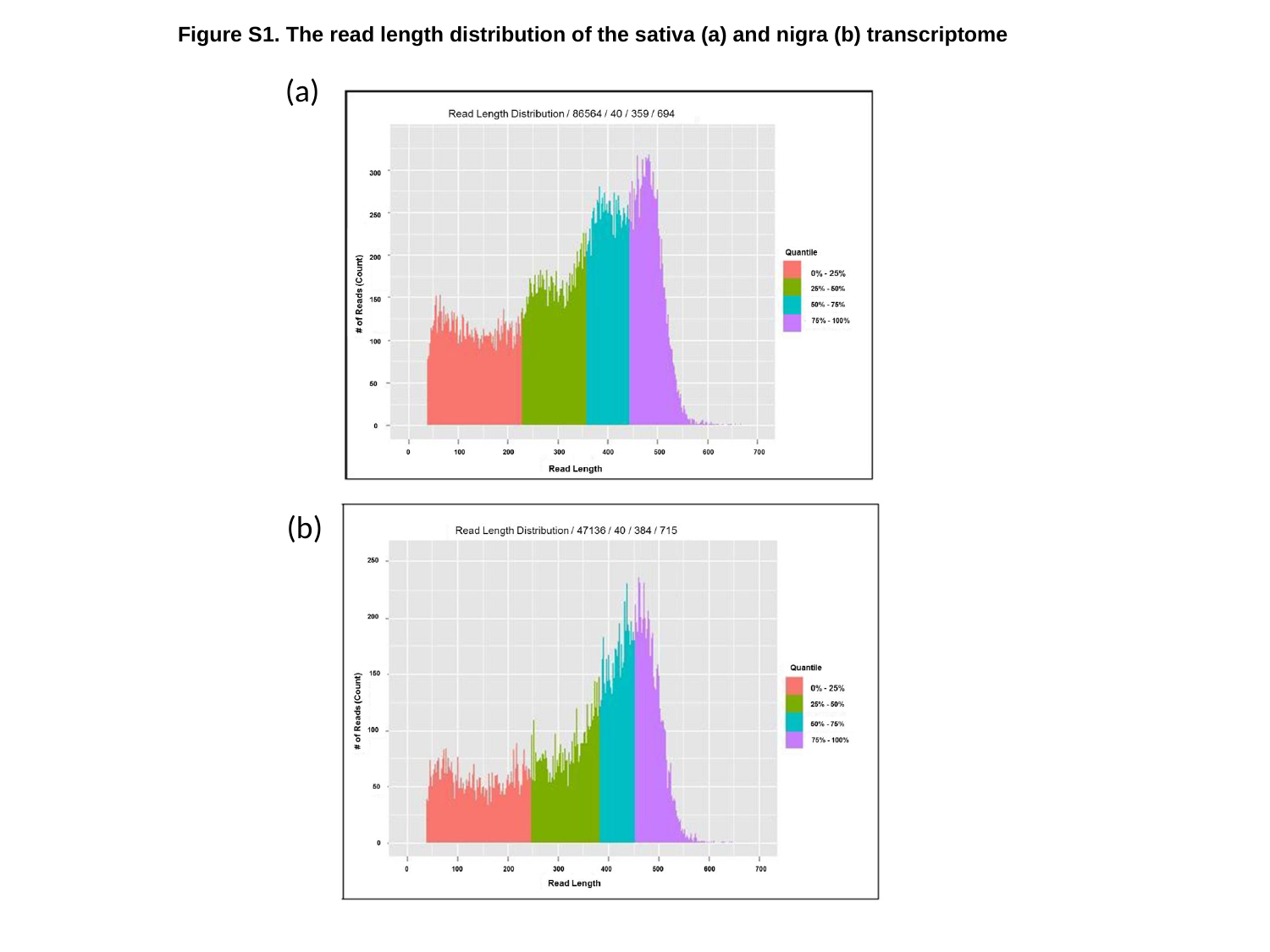

Figure S1. The read length distribution of the sativa (a) and nigra (b) transcriptome
(a)
(b)

Supplement: Supplementary File 1 [file genes-06-01164-s001.zip › Figure S1.pptx]
